# Supplementary material for: Excessive neutrophil recruitment promotes typical T-helper 17 responses in Coronavirus disease 2019 patients
Source: PLoS One. 2022 Aug 18;17(8):e0273186. doi: 10.1371/journal.pone.0273186 (PMC9387804; doi:10.1371/journal.pone.0273186)
Supplement: S1 Table — (DOCX) [file pone.0273186.s001.docx]

| **Patient ID** | **Sex** | **Age** |
| --- | --- | --- |
|  |  |  |
| LN14202 | Male | 48.72 |
| LN10001 | Male | 48.72 |
| LN10002 | Female | 48.72 |
| LN10003 | Male | 48.72 |
| LN14208 | Male | 82 |
| LN14209 | Female | 44 |
| LN14231 | Male | 52 |
| LN14248 | Female | 48 |
| LN14249 | Male | 60 |
| LN14255 | Male | 50 |
| LN14256 | Male | 53 |
| LN14257 | Male | 64 |
| LN14258 | Male | 49.16 |
| LN14259 | Male | 48.72 |
| LN14260 | Male | 65.99 |
| LN14261 | Female | 63.32 |
| LN14287 | Female | 47 |
| LN14288 | Male | 39 |
| LN14298 | Male | 28 |
| LN14302 | Male | 48.72 |
| LN14303 | Male | 39 |
| LN14304 | Female | 47 |
| LN14321 | Female | 48.72 |
| LN14322 | Female | 48.72 |
| LN14327 | Male | 60.73 |
| LN14328 | Male | 62 |
| LN14351 | Male | 64.99 |
| LN14352 | Male | 28.49 |
| LN14354 | Male | 56 |
| LN14355 | Female | 45 |
| LN14356 | Male | 59.99 |
| LN14411 | Male | 56 |
| LN14412 | Female | 33 |
| LN14413 | Male | 65 |
| LN14414 | Female | 73 |
| LN14478 | Male | 45 |
| LN14479 | Male | 67 |
| LN14449 | Female | 40 |
| LN14447 | Male | 40 |
| LN14448 | Male | 40 |
| LN14446 | Male | 53 |
| LN14568 | Male | 45 |
| LN14567 | Female | 37 |
|  |  |  |
