# Supplementary material for: Excessive neutrophil recruitment promotes typical T-helper 17 responses in Coronavirus disease 2019 patients
Source: PLoS One. 2022 Aug 18;17(8):e0273186. doi: 10.1371/journal.pone.0273186 (PMC9387804; doi:10.1371/journal.pone.0273186)
Supplement: S2 Table — (DOCX) [file pone.0273186.s002.docx]

| **Patient ID** | **WBC Count**  **(x 10^9^/L)** | **RBC Count**  **(x10^12^/L)** | **HGB Count (g/dl)** | **HCT**  **(%)** | **MCV**  **(fl)** | **MCH**  **(pg)** | **MCHC (g/dl)** | **RCDWC (%)** | **RCDW**  **SD**  **(fl)** | **Platelets (x 10^9^/L)** |
| --- | --- | --- | --- | --- | --- | --- | --- | --- | --- | --- |
|  |  |  |  |  |  |  |  |  |  |  |
| LN14202 | 17.8 | 2.76 | 8 | 25.6 | 93.1 | 28.9 | 31.2 | 17.2 |  | 144 |
| LN10001 | 3.41 | 4.58 | 13.6 | 47.5 | 103.7 | 29.7 | 28.6 | 13.4 | 51.5 | 230 |
| LN10002 | 8.67 | 4.95 | 13.6 | 50 | 101 | 27.5 | 27.2 | 18.1 | 64.9 | 109 |
| LN10003 | 7.92 | 3.67 | 11 | 38.1 | 103.8 | 30 | 28.9 | 15.4 | 59.7 | 180 |
| LN14208 | 8.86 | 4.62 | 12.2 | 42.5 | 92 | 26.4 | 28.7 | 14.4 | 47.8 | 77 |
| LN14209 | 9.2 | 1.47 | 4.3 | 14.2 | 97 | 29.2 | 30 | 17.7 |  | 333 |
| LN14231 | 7.41 | 3.23 | 12.22 | 37.9 | 75.9 | 26.7 | 31 | 16.4 |  | 313.5 |
| LN14248 | 9.92 | 4.41 | 14.4 | 49.7 | 112.7 | 32.7 | 29 | 14.4 | 60.6 | 540 |
| LN14249 | 15.71 | 4.59 | 14.3 | 44.3 | 96.5 | 31.2 | 32.3 | 13.5 | 47.9 | 533 |
| LN14255 | 5.07 | 3.79 | 11.7 | 44.7 | 117.9 | 30.9 | 26.2 | 16.6 | 73.2 | 174 |
| LN14256 | 10.2 | 5.1 | 13.5 | 47.5 | 93.1 | 26.5 | 28.4 | 15.7 | 53.9 | 470 |
| LN14257 | 11.57 | 4.29 | 12.1 | 46 | 107.2 | 28.2 | 26.3 | 17.6 | 67.3 | 54 |
| LN14258 | 11.34 | 4.58 | 12.8 | 42.4 | 92.6 | 27.9 | 30.2 | 13.9 | 46.5 | 480 |
| LN14259 | 10.73 | 2.4 | 6.7 | 24.3 | 101.3 | 27.9 | 27.6 | 15.5 | 57.8 | 232 |
| LN14260 | 3.35 | 2.83 | 7.8 | 28 | 98.9 | 27.6 | 27.9 | 15.7 | 56.2 | 202 |
| LN14261 | 2.16 | 4.07 | 11.5 | 38.3 | 94.1 | 28.3 | 30 | 14.6 | 49.57 | 140 |
| LN14287 | 14.7 | 3.2 | 10.7 | 34 | 109.3 | 34.7 | 31.4 | 23.7 |  | 180 |
| LN14288 | 12.9 | 2.43 | 8.1 | 16.7 | 119.6 | 33.3 | 29.8 | 17.7 |  | 155 |
| LN14298 | 6.3 | 5.32 | 15.4 | 47.5 | 89.3 | 28.9 | 32.4 | 17.2 |  | 211 |
| LN14302 | 4.7 | 1.27 | 3.6 | 11.1 | 88.1 | 28.3 | 32.4 | 13.8 |  | 191 |
| LN14321 | 16.47 | 5.46 | 16 | 54 | 98.9 | 29.3 | 29.6 | 18.3 | 63.6 | 103 |
| LN14322 | 9.28 | 4.91 | 14.8 | 50.9 | 103.7 | 30.1 | 29.1 | 14.8 | 57.2 | 123 |
| LN14327 | 5.15 | 4.83 | 14.9 | 52.3 | 108.3 | 30.8 | 28.5 | 12.6 | 49.9 | 221 |
| LN14351 | 6.01 | 3.2 | 10.1 | 32.2 | 100.6 | 31.6 | 31.4 | 16.9 | 60.5 | 49 |
| LN14352 | 8.21 | 4.95 | 14.3 | 43.6 | 88.1 | 28.9 | 32.8 | 13.6 | 43.1 | 365 |
| LN14354 | 11.47 | 6.03 | 13.6 | 47.9 | 79.4 | 22.6 | 28.4 | 18.7 | 49.9 | 315 |
| LN14355 | 9.5 | 4.51 | 13 | 42.3 | 93.8 | 28.8 | 30.7 | 14.3 | 49 | 402 |
| LN14356 | 8.76 | 5.22 | 15.8 | 46.5 | 89.1 | 30.3 | 34 | 14.5 | 46.5 | 140 |
| LN14411 | 11.42 | 5.47 | 15.4 | 49.4 | 90.3 | 28.2 | 31.2 | 14.3 | 46.9 | 202 |
| LN14412 | 12.27 | 4.2 | 10.4 | 37.5 | 89.3 | 24.8 | 27.7 | 35 | 109.9 | 240 |
| LN14413 | 14.69 | 4.34 | 11.8 | 35.8 | 82.5 | 27.2 | 33 | 17.4 | 50.1 | 339 |
| LN14414 | 33.78 | 4.69 | 12.7 | 45.1 | 96.2 | 27.1 | 28.2 | 17.2 | 58.4 | 197 |
| LN14478 | 8.05 | 4.45 | 12.2 | 38.4 | 86.3 | 27.4 | 31.8 | 15.9 | 49.3 | 352 |
| LN14479 | 22.81 | 3.11 | 9.7 | 26.8 | 86.2 | 31.2 | 36.2 | 14.6 | 43.8 | 415 |
| LN14449 | 12.05 | 6.48 | 16.8 | 56.3 | 86.9 | 25.9 | 29.8 | 19.6 | 54.5 | 122 |
| LN14447 | 10.17 | 4.66 | 14 | 49.5 | 106.2 | 30 | 28.3 | 16.1 | 63 | 261 |
| LN14448 | 12.87 | 3.54 | 9 | 32.3 | 91.2 | 25.4 | 27.9 | 24.6 | 74.9 | 485 |
| LN14446 | 4.27 | 3.92 | 13.1 | 44.5 | 113.5 | 33.4 | 29.4 | 16.8 | 71.3 | 158 |
| LN14568 | 12.3 | 8.42 | 13.9 | 51.5 | 61.2 | 16.5 | 26.9 | 17.7 | 36.6 | 187 |
| LN14567 | 15.4 | 4.17 | 8.8 | 33.5 | 80.4 | 21.1 | 26.2 | 19.1 | 53.7 | 219 |
|  |  |  |  |  |  |  |  |  |  |  |
