# Supplementary material for: Excessive neutrophil recruitment promotes typical T-helper 17 responses in Coronavirus disease 2019 patients
Source: PLoS One. 2022 Aug 18;17(8):e0273186. doi: 10.1371/journal.pone.0273186 (PMC9387804; doi:10.1371/journal.pone.0273186)
Supplement: S3 Table — (DOCX) [file pone.0273186.s003.docx]

| **Patient ID** | **Neut**  **(x 10^9^/L)** | **Neut (%)** | **Lymph (x 10^9^/L)** | **Lymph (%)** | **Eosino**  **(x 10^9^/L)** | **Eosino (%)** | **Mono (x 10^9^/L)** | **Mono (%)** | **Baso**  **(x 10^9^/L)** | **Baso (%)** | **IG**  **(x 10^9^/L)** | **IG**  **(%)** |
| --- | --- | --- | --- | --- | --- | --- | --- | --- | --- | --- | --- | --- |
|  |  |  |  |  |  |  |  |  |  |  |  |  |
| LN14202 | 13.4 | 74.9 | 2.9 | 16.3 | 0 | 0 | 1.5 | 8 | 0 | 0 |  |  |
| LN10001 | 1.5 | 44 | 1.64 | 48.1 | 0 | 0 | 0.16 | 4.7 | 0.01 | 0.3 | 0.1 | 2.9 |
| LN10002 | 4.21 | 48.7 | 1.46 | 16.8 | 0.01 | 0.1 | 2.04 | 23.5 | 0.07 | 0.8 | 0.88 | 10.1 |
| LN10003 | 6.67 | 84.2 | 0.69 | 8.7 | 0 | 0 | 0.22 | 2.8 | 0.02 | 0.3 | 0.32 | 4 |
| LN14208 | 7 | 79 | 0.59 | 6.7 | 0.01 | 0.1 | 0.56 | 6.3 | 0.03 | 0.3 | 0.67 | 7.6 |
| LN14209 | 2.5 | 27.6 | 5.7 | 61.2 | 0 | 0 | 1 | 11.2 | 0 | 0 |  |  |
| LN14231 | 4.83 | 65.18 | 1.48 | 19.97 | 0.03 | 0.4 | 1.07 | 14.44 | 0.01 | 0.13 |  |  |
| LN14248 | 6.87 | 69.3 | 2.32 | 23.4 | 0.02 | 0.2 | 0.61 | 6.1 | 0.02 | 0.2 | 0.08 | 0.8 |
| LN14249 | 13.74 | 87.4 | 0.99 | 6.3 | 0 | 0 | 0.72 | 4.6 | 0.04 | 0.3 | 0.22 | 1.4 |
| LN14255 | 3.65 | 71.9 | 0.73 | 14.4 | 0.02 | 0.4 | 0.14 | 2.8 | 0.14 | 2.8 | 0.39 | 7.7 |
| LN14256 | 9.24 | 90.5 | 0.2 | 2 | 0.03 | 0.3 | 0.07 | 0.7 | 0.03 | 0.3 | 0.63 | 6.2 |
| LN14257 | 10.15 | 87.7 | 0.43 | 3.7 | 0.07 | 0.6 | 0.08 | 0.7 | 0.1 | 0.9 | 0.74 | 6.4 |
| LN14258 | 9.54 | 84.1 | 0.96 | 8.5 | 0.01 | 0.1 | 0.66 | 5.8 | 0.03 | 0.3 | 0.14 | 1.2 |
| LN14259 | 8.58 | 80 | 0.68 | 6.3 | 0.26 | 2.4 | 0.17 | 1.6 | 0.02 | 0.2 | 1.02 | 9.5 |
| LN14260 | 1.98 | 59 | 0.79 | 23.6 | 0.2 | 6 | 0.05 | 1.5 | 0.04 | 1.2 | 0.29 | 8.7 |
| LN14261 | 1.16 | 53.7 | 0.64 | 29.6 | 0.04 | 1.9 | 0.08 | 3.7 | 0.02 | 0.9 | 0.22 | 10.2 |
| LN14287 | 12.1 | 82.1 | 1.7 | 11.3 | 0 | 0 | 0.9 | 6.6 | 0 | 0 |  |  |
| LN14288 | 7.1 | 55.4 | 3.9 | 30.2 | 0 | 0 | 1.9 | 14.4 | 0 | 0 |  |  |
| LN14298 | 3 | 47.3 | 1.2 | 19.45 | 0 | 0 | 2.1 | 33.3 | 0 | 0 |  |  |
| LN14302 | 1.8 | 38.2 | 2.3 | 48.6 | 0 | 0 | 0.6 | 13.2 | 0 | 0 |  |  |
| LN14321 | 11.24 | 68.3 | 3.19 | 19.4 | 0 | 0 | 0.6 | 3.6 | 0.22 | 1.3 | 1.22 | 7.4 |
| LN14322 | 7.26 | 78.2 | 0.85 | 9.2 | 0 | 0 | 0.62 | 6.7 | 0.02 | 0.2 | 0.53 | 5.7 |
| LN14327 | 3.2 | 62.1 | 1.41 | 27.4 | 0.07 | 1.4 | 0.28 | 5.4 | 0.02 | 0.4 | 0.17 | 3.3 |
| LN14351 | 2.95 | 49 | 2.24 | 37.3 | 0 | 0 | 0.49 | 8.2 | 0.02 | 0.3 | 0.31 | 5.2 |
| LN14352 | 7.17 | 87.3 | 0.82 | 10 | 0 | 0 | 0.17 | 2.1 | 0.01 | 0.1 | 0.04 | 0.5 |
| LN14354 | 9.04 | 78.9 | 1.55 | 13.5 | 0.12 | 1 | 0.6 | 5.2 | 0.07 | 0.6 | 0.09 | 0.8 |
| LN14355 | 5.97 | 62.9 | 3.08 | 32.4 | 0.05 | 0.5 | 0.35 | 3.7 | 0.01 | 0.1 | 0.04 | 0.4 |
| LN14356 | 6.95 | 79.3 | 1.01 | 11.5 | 0 | 0 | 0.75 | 8.6 | 0.01 | 0.1 | 0.04 | 0.5 |
| LN14411 | 9.21 | 80.6 | 1.45 | 12.7 | 0 | 0 | 0.65 | 5.7 | 0.03 | 0.3 | 0.08 | 0.7 |
| LN14412 | 11.38 | 92.8 | 0.3 | 2.4 | 0 | 0 | 0.15 | 1.2 | 0.11 | 0.9 | 0.33 | 2.7 |
| LN14413 | 12.46 | 84.8 | 0.65 | 4.4 | 0.01 | 0.1 | 1.14 | 7.8 | 0.03 | 0.2 | 0.4 | 2.7 |
| LN14414 | 29.8 | 88.7 | 1.93 | 5.7 | 0.27 | 0.8 | 0.8 | 2.4 | 0.12 | 0.4 | 0.68 | 2 |
| LN14478 | 7.12 | 88.5 | 0.69 | 8.6 | 0 | 0 | 0.09 | 1.1 | 0.02 | 0.2 | 0.13 | 1.6 |
| LN14479 | 21.2 | 92.9 | 0.89 | 3.9 | 0 | 0 | 0.23 | 1 | 0.04 | 0.2 | 0.45 | 2 |
| LN14449 | 10.4 | 86.3 | 0.65 | 5.4 | 0.01 | 0.1 | 0.17 | 1.4 | 0.2 | 1.7 | 0.62 | 5.1 |
| LN14447 | 8.5 | 83.5 | 1.19 | 11.7 | 0.08 | 0.8 | 0.23 | 2.3 | 0.05 | 0.5 | 0.12 | 1.2 |
| LN14448 | 11.97 | 93.1 | 0.05 | 0.4 | 0.03 | 0.2 | 0.48 | 3.7 | 0.03 | 0.2 | 0.31 | 2.4 |
| LN14446 | 2.75 | 64.5 | 1.02 | 23.9 | 0.01 | 0.2 | 0.39 | 9.1 | 0.03 | 0.7 | 0.07 | 1.6 |
| LN14568 | 4.5 | 36.9 | 5.4 | 43.2 |  |  | 2.4 | 19.9 |  |  |  |  |
| LN14567 | 11.1 | 72.2 | 2.9 | 18.9 |  |  | 1.4 | 8.9 |  |  |  |  |
|  |  |  |  |  |  |  |  |  |  |  |  |  |
