# Supplementary material for: Excessive neutrophil recruitment promotes typical T-helper 17 responses in Coronavirus disease 2019 patients
Source: PLoS One. 2022 Aug 18;17(8):e0273186. doi: 10.1371/journal.pone.0273186 (PMC9387804; doi:10.1371/journal.pone.0273186)
Supplement: S4 Table — (DOCX) [file pone.0273186.s004.docx]

| **Patient ID** | **CD3^+^ (%)** | **CD3^+^**  **(cells/μl)** | **CD8^+^ (%)** | **CD8^+^ (cells/μl)** | **CD4^+^ (%)** | **CD4^+^ (cells/μl)** | **CD16^+^/CD56^+^/CD16^+^CD56^+^ (%)** | **CD16^+^/CD56^+^/CD16^+^CD56^+^ (cells/μl)** | **CD19^+^ (%)** | **CD19^+^ (cells/μl)** | **CD4+/CD8+ Ratio** |
| --- | --- | --- | --- | --- | --- | --- | --- | --- | --- | --- | --- |
|  |  |  |  |  |  |  |  |  |  |  |  |
| LN14202 | 37 | 1062 | 35 | 1012 | 2 | 46 | 75 | 2190 | 5 | 142 | 0.05 |
| LN10001 | 63 | 1025 | 34 | 562 | 22 | 364 | 3 | 42 | 12 | 189 | 0.65 |
| LN10002 | 11 | 164 | 5 | 78 | 6 | 83 | 1 | 17 | 56 | 822 | 1.07 |
| LN10003 | 39 | 269 | 19 | 131 | 12 | 85 | 7 | 51 | 17 | 120 | 0.65 |
| LN14208 | 31 | 1373 | 5 | 229 | 3 | 143 | 2 | 94 | 9 | 420 | 0.62 |
| LN14209 | 47 | 2640 | 28 | 1558 | 18 | 1026 | 38 | 2147 | 31 | 1769 | 0.66 |
| LN14231 | 45 | 666 | 34 | 500 | 6 | 95 | 32 | 479 | 34 | 507 | 0.19 |
| LN14248 | 74 | 1710 | 69 | 1609 | 1 | 34 | 2 | 43 | 3 | 63 | 0.02 |
| LN14249 | 51 | 504 | 17 | 171 | 31 | 308 | 23 | 224 | 43 | 421 | 1.8 |
| LN14255 | 22 | 162 | 20 | 144 | 2 | 16 | 71 | 519 | 2 | 18 | 0.11 |
| LN14256 | 6 | 13 | 0 | 1 | 2 | 4 | 15 | 31 | 4 | 7 | 4.45 |
| LN14257 | 24 | 104 | 2 | 9 | 15 | 65 | 15 | 66 | 3 | 12 | 7.64 |
| LN14258 | 47 | 455 | 39 | 373 | 3 | 29 | 31 | 300 | 6 | 55 | 0.08 |
| LN14259 | 35 | 234 | 28 | 191 | 0 | 2 | 69 | 464 | 5 | 34 | 0.01 |
| LN14260 | 20 | 160 | 16 | 128 | 1 | 6 | 26 | 206 | 4 | 36 | 0.05 |
| LN14261 | 82 | 536 | 80 | 521 | 0 | 2 | 2 | 12 | 2 | 14 | 0 |
| LN14287 | 70.54 | 1172 | 14.61 | 243 | 28.98 | 481 | 2 | 38 | 24 | 392 | 1.98 |
| LN14288 | 54.53 | 2385 | 48.7 | 2130 | 1.98 | 87 | 4 | 166 | 43 | 1871 | 0.04 |
| LN14298 | 59.81 | 735 | 22.21 | 273 | 32.2 | 396 | 6 | 69 | 30 | 364 | 1.45 |
| LN14302 | 48.27 | 1102 | 25.3 | 578 | 19.73 | 451 | 10 | 230 | 38 | 871 | 0.78 |
| LN14303 | 80.66 | 1340 | 14.68 | 244 | 34.29 | 570 | 5 | 80 | 12 | 193 | 2.33 |
| LN14304 | 74.38 | 3253 | 61.28 | 2680 | 6.42 | 281 | 3 | 135 | 21 | 898 | 0.1 |
| LN14321 | 71.38 | 617 | 27.97 | 242 | 33.45 | 289 | 5 | 46 | 18 | 157 | 1.19 |
| LN14322 | 68.84 | 2204 | 29.09 | 931 | 37.31 | 1194 | 3 | 104 | 28 | 886 | 1.28 |
| LN14327 | 32.3 | 460 | 0.54 | 8 | 0.42 | 6 | 6 | 79 | 6 | 87 | 0.78 |
| LN14351 | 62 | 1380 | 21 | 480 | 33 | 740 | 3 | 75 | 29 | 642 | 1.54 |
| LN14352 | 76 | 626 | 70 | 571 | 1 | 9 | 2 | 20 | 21 | 172 | 0.02 |
| LN14354 | 55 | 855 | 20 | 305 | 14 | 255 | 8 | 124 | 32 | 494 | 0.74 |
| LN14355 | 66 | 2021 | 48 | 1478 | 12 | 356 | 2 | 52 | 15 | 473 | 0.24 |
| LN14356 | 41 | 419 | 23 | 236 | 13 | 132 | 3 | 27 | 52 | 531 | 0.56 |
| LN14411 | 47.09 | 682 | 28.51 | 413 | 9.54 | 138 | 6 | 93 | 40 | 572 | 0.33 |
| LN14412 | 87.8 | 259 | 26.22 | 77 | 43.79 | 129 | 1 | 2 | 12 | 34 | 1.67 |
| LN14413 | 48.75 | 315 | 39.19 | 254 | 7.57 | 49 | 6 | 38 | 45 | 288 | 0.19 |
| LN14414 | 54.91 | 1058 | 28.11 | 542 | 20.63 | 397 | 4 | 79 | 40 | 764 | 0.73 |
| LN14478 | 8 | 55 | 6 | 41 | 2 | 11 | 2 | 13 | 11 | 76 | 0.26 |
| LN14479 | 17 | 153 | 27 | 240 | 2 | 13 | 61 | 543 | 2 | 16 | 0.06 |
| LN14449 | 48 | 315 | 8 | 52 | 34 | 222 |  |  |  |  | 4.32 |
| LN14447 | 70 | 838 | 31 | 372 | 38 | 451 |  |  |  |  | 1.21 |
| LN14448 | 81 | 42 | 25 | 13 | 38 | 19 |  |  |  |  | 1.5 |
| LN14568 | 67 | 3554 | 47 | 2482 | 13 | 669 | 3 | 147 | 29 | 1535 | 0.27 |
| LN14567 | 74 | 2165 | 54 | 1581 | 13 | 388 | 5 | 146 | 17 | 495 | 0.25 |
|  |  |  |  |  |  |  |  |  |  |  |  |
