# Supplementary material for: Excessive neutrophil recruitment promotes typical T-helper 17 responses in Coronavirus disease 2019 patients
Source: PLoS One. 2022 Aug 18;17(8):e0273186. doi: 10.1371/journal.pone.0273186 (PMC9387804; doi:10.1371/journal.pone.0273186)
Supplement: S5 Table — (DOCX) [file pone.0273186.s005.docx]

| **Patient ID** | **CRP Status** |
| --- | --- |
|  |  |
| LN14202 | Positive |
| LN14208 | Positive |
| LN14209 | Positive |
| LN14231 | Positive |
| LN14248 | Positive |
| LN14249 | Positive |
| LN14255 | Positive |
| LN14256 | Positive |
| LN14257 | Positive |
| LN14258 | Positive |
| LN14259 | Positive |
| LN14260 | Negative |
| LN14261 | Negative |
| LN14287 | Positive |
| LN14288 | Positive |
| LN14298 | Positive |
| LN14303 | Positive |
| LN14304 | Positive |
| LN14321 | Positive |
| LN14322 | Positive |
| LN14327 | Negative |
| LN14328 | Positive |
| LN14351 | Positive |
| LN14352 | Positive |
| LN14354 | Negative |
| LN14356 | Positive |
| LN14411 | Positive |
| LN14412 | Positive |
| LN14413 | Positive |
| LN14414 | Positive |
| LN14478 | Positive |
| LN14479 | Positive |
| LN14449 | Positive |
| LN14447 | Negative |
| LN14448 | Positive |
|  |  |
