# Supplementary material for: Excessive neutrophil recruitment promotes typical T-helper 17 responses in Coronavirus disease 2019 patients
Source: PLoS One. 2022 Aug 18;17(8):e0273186. doi: 10.1371/journal.pone.0273186 (PMC9387804; doi:10.1371/journal.pone.0273186)
Supplement: S6 Table — (DOCX) [file pone.0273186.s006.docx]

| **Patient ID** | **Sample** | **IL-17A** | **IFN-γ** | **TNF-α** | **IL-10** | **IL-6** | **IL-4** | **IL-2** |
| --- | --- | --- | --- | --- | --- | --- | --- | --- |
|  |  |  |  |  |  |  |  |  |
| LN14202 | Hospitalised | 0 | 0 | 0 | 16.52 | 96.31 | 0 | 0 |
| LN10001 | Hospitalised | 0 | 0 | 0 | 0 | 0 | 0 | 0 |
| LN10002 | Hospitalised | 0 | 0 | 0 | 31.24 | 751.79 | 0 | 0 |
| LN14208 | Hospitalised | 0 | 42.53 | 0 | 30.01 | 445.26 | 0 | 0 |
| LN14209 | Hospitalised | 0 | 0 | 0 | 6.51 | 168.98 | 0 | 0 |
| LN14231 | Hospitalised | 0 | 0 | 0 | 3.9 | 159.88 | 0 | 0 |
| LN14248 | Hospitalised | 0 | 0 | 0 | 0 | 61.75 | 0 | 0 |
| LN14249 | Hospitalised | 0 | 0 | 0 | 38.28 | 853.11 | 0 | 0 |
| LN14255 | Hospitalised | 0 | 0 | 120.52 | 84.94 | 17737.31 | 0 | 0 |
| LN14256 | Hospitalised | 0 | 0 | 0 | 32.83 | 124.9 | 0 | 0 |
| LN14257 | Hospitalised | 70.23 | 0 | 422.9 | 512.17 | 424802.4 | 0 | 0 |
| LN14259 | Hospitalised | 319.26 | 0 | 2088.4 | 4842.09 | 12.64 | 0 | 0 |
| LN14260 | Hospitalised | 0 | 0 | 726.79 | 0 | 0 | 0 | 0 |
| LN14261 | Hospitalised | 0 | 0 | 177.19 | 0 | 2655.12 | 0 | 0 |
| LN14287 | Hospitalised | 0 | 0 | 0 | 0 | 625.72 | 0 | 0 |
| LN14288 | Hospitalised | 0 | 0 | 0 | 20.46 | 282.63 | 0 | 0 |
| LN14298 | Hospitalised | 0 | 0 | 0 | 0 | 0 | 0 | 0 |
| LN14302 | Hospitalised | 0 | 0 | 0 | 0 | 868.83 | 0 | 0 |
| LN14303 | Hospitalised | 0 | 0 | 60.26 | 33.48 | 100525.3 | 0 | 0 |
| LN14304 | Hospitalised | 0 | 0 | 0 | 0 | 306.16 | 0 | 0 |
| LN14321 | Hospitalised | 0 | 0 | 14.43 | 213.4 | 3294.17 | 0 | 0 |
| LN14322 | Hospitalised | 0 | 0 | 0 | 34.14 | 3549.25 | 0 | 0 |
| LN14327 | Hospitalised | 0 | 0 | 0 | 0 | 229.92 | 0 | 0 |
| LN14328 | Hospitalised | 0 | 0 | 0 | 407.36 | 1295.51 | 0 | 0 |
| LN14351 | Hospitalised | 0 | 229.26 | 0 | 13.36 | 3214.29 | 0 | 0 |
| LN14352 | Hospitalised | 0 | 0 | 93.24 | 0 | 73.58 | 0 | 0 |
| LN14354 | Hospitalised | 0 | 0 | 32.23 | 0 | 0 | 0 | 0 |
| LN14356 | Hospitalised | 0 | 0 | 46.54 | 0 | 1201856 | 0 | 0 |
| LN14411 | Hospitalised | 85.57 | 0 | 0 | 54.27 | 7154 | 2314.98 | 0 |
| LN14412 | Hospitalised | 0 | 61.3 | 0 | 0 | 1233.26 | 0 | 0 |
| LN14413 | Hospitalised | 263.86 | 20.1 | 0 | 51 | 513.31 | 0 | 0 |
| LN14414 | Hospitalised | 287.97 | 13.74 | 0 | 60.62 | 1295.51 | 94.69 | 0 |
| LN14478 | Hospitalised | 0 | 0 | 0 | 0 | 311.11 | 0 | 0 |
| LN14479 | Hospitalised | 323.89 | 52.33 | 0 | 76.44 | 1097.95 | 0 | 0 |
| LN14449 | Hospitalised | 0 | 0 | 0 | 127.45 | 1036.99 | 0 | 0 |
| LN14447 | Hospitalised | 0 | 0 | 24.23 | 0 | 29340.72 | 0 | 0 |
| LN14448 | Hospitalised | 0 | 0 | 0 | 11.2 | 18447.92 | 0 | 0 |
| LN14568 | Hospitalised | 0 | 0 | 0 | 30.01 | 522.06 | 0 | 0 |
| LN14567 | Hospitalised | 0 | 1482 | 0 | 60.11 | 0 | 0 | 0 |
|  |  |  |  |  |  |  |  |  |
