# Supplementary material for: Excessive neutrophil recruitment promotes typical T-helper 17 responses in Coronavirus disease 2019 patients
Source: PLoS One. 2022 Aug 18;17(8):e0273186. doi: 10.1371/journal.pone.0273186 (PMC9387804; doi:10.1371/journal.pone.0273186)
Supplement: S7 Table — (DOCX) [file pone.0273186.s007.docx]

| **Patient ID** | **Sample** | **IL-17A** | **IFN-γ** | **TNF-α** | **IL-10** | **IL-6** | **IL-4** | **IL-2** |
| --- | --- | --- | --- | --- | --- | --- | --- | --- |
|  |  |  |  |  |  |  |  |  |
| LN210016 | Control | 0 | 0 | 0 | 0 | 0 | 0 | 0 |
| LN14374 | Control | 0 | 0 | 0 | 0 | 291.8 | 0 | 0 |
| LN210009 | Control | 0 | 0 | 0 | 0 | 0 | 0 | 0 |
| LN210013 | Control | 0 | 0 | 0 | 0 | 0 | 0 | 0 |
| LN210011 | Control | 0 | 0 | 0 | 0 | 2477.11 | 0 | 0 |
| LN210017 | Control | 0 | 0 | 0 | 0 | 0 | 0 | 0 |
| LN210018 | Control | 0 | 0 | 0 | 0 | 0 | 0 | 0 |
| LN210010 | Control | 0 | 0 | 0 | 0 | 147.69 | 0 | 0 |
| LN14522 | Control | 0 | 0 | 0 | 0 | 101.17 | 0 | 0 |
| LN210008 | Control | 0 | 0 | 0 | 0 | 0 | 0 | 0 |
| LN210019 | Control | 0 | 0 | 0 | 0 | 0 | 0 | 0 |
| LN14529 | Control | 0 | 0 | 0 | 0 | 0 | 0 | 0 |
| LN14535 | Control | 0 | 0 | 0 | 0 | 0 | 0 | 0 |
| LN14520 | Control | 0 | 0 | 0 | 0 | 0 | 0 | 0 |
| LN210014 | Control | 0 | 0 | 0 | 0 | 0 | 0 | 0 |
| LN14383 | Control | 0 | 0 | 0 | 0 | 0 | 0 | 0 |
| LN210007 | Control | 0 | 0 | 0 | 0 | 0 | 0 | 0 |
| LN210004 | Control | 0 | 0 | 0 | 0 | 0 | 0 | 0 |
|  |  |  |  |  |  |  |  |  |
