# Supplementary material for: Excessive neutrophil recruitment promotes typical T-helper 17 responses in Coronavirus disease 2019 patients
Source: PLoS One. 2022 Aug 18;17(8):e0273186. doi: 10.1371/journal.pone.0273186 (PMC9387804; doi:10.1371/journal.pone.0273186)
Supplement: S8 Table — (DOCX) [file pone.0273186.s008.docx]

| **Variable** | **COVID-19 patients with low NLR**  **Median (Q1, Q3)** | | **COVID-19 patients with high NLR**  **Median (Q1, Q3)** | ***p*** |
| --- | --- | --- | --- | --- |
|  |  | |  |  |
| *n* | 24 | | 16 |  |
| Age | 48.72135 (44.5, 56) | | 48.94067 (40, 64.5) | 0.5800 |
| Sex: |  | |  | 0.729 |
| Female, *n* (%) | 8 (33.33) | | 4 (25.00) |  |
| Male, *n* (%) | 16 (66.67) | | 12 (75.00) |  |
| Red Blood Cell Count (x 10^12^/L) | 4.29 (3.20, 5.085) | | 4.515 (3.935, 4.80) | 0.6586 |
| Haemoglobin Count (g/dl) | 13.05 (9.45, 14.20) | | 12.20 (10.70, 13.90) | 0.8037 |
| Haematocrit (%) | 44.6 (32.85, 49.45) | | 42.45 (36.65, 45.55) | 0.6991 |
| Mean Corpuscular Volume (fl) | 97.95 (89.20, 107.25) | | 93.95 (88.60, 103.70) | 0.2351 |
| Mean Corpuscular Haemoglobin (pg) | 29.05 (27.9, 30.85) | | 27.65 (26.45, 29.45) | 0.0683 |
| Mean Corpuscular Haemoglobin Concentration (g/dl) | 29.70 (28.35, 31.20) | | 29.00 (28.05, 32.05) | 0.9670 |
| Red Cell Distribution Width Coefficient (%) | 16.70 (14.45, 17.70) | | 15.60 (14.50, 17.50) | 0.7299 |
| Red Cell Distribution Width Standard Deviation (fl) | 53.70 (49.57, 63.00) | | 54.20 (47.85, 59.05) | 0.7594 |
| Platelets (x 10^9^/L) | 196.50 (149.50, 245.50) | | 289.50 (151.50, 442.50) | 0.1324 |
|  |  |  | |  |
