# Supplementary figures and images for: Excessive neutrophil recruitment promotes typical T-helper 17 responses in Coronavirus disease 2019 patients
Source: PLoS One. 2022 Aug 18;17(8):e0273186. doi: 10.1371/journal.pone.0273186 (PMC9387804; doi:10.1371/journal.pone.0273186)

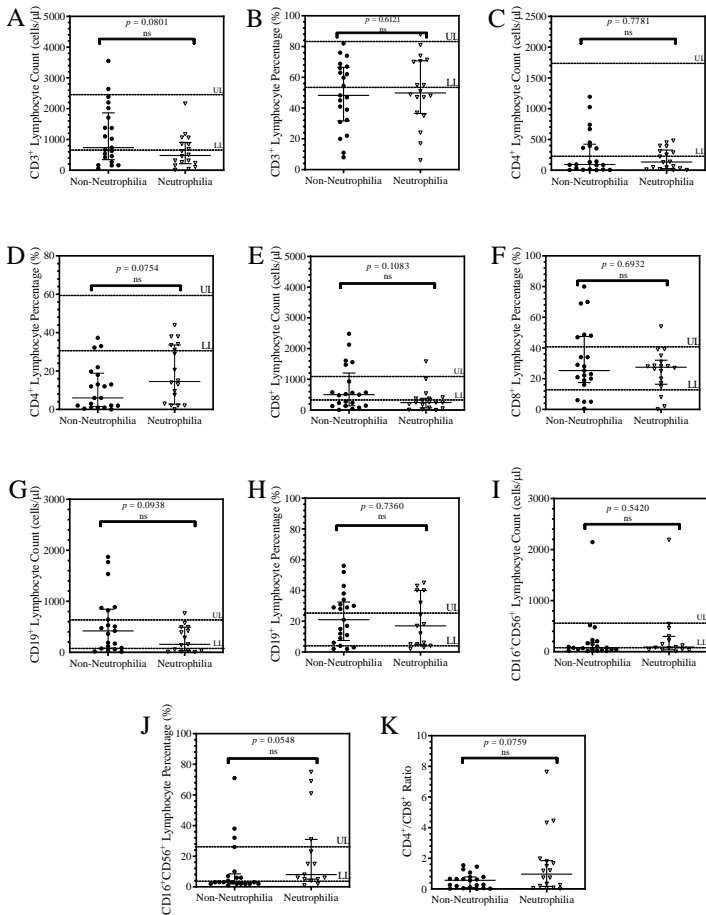

Supplement: S1 Fig — (PDF) [file pone.0273186.s009.pdf]
